# Clinical Analysis of the HBV Infection Status of 135 Patients with Diffuse Large B Cell Lymphoma Treated with R-CHOP or CHOP/CHOP-Like Chemotherapy

**DOI:** 10.1371/journal.pone.0129064

**Published:** 2015-06-08

**Authors:** Wei Guo, Wenxian Zhang, Chunshui Liu, Yuanyuan Song, Ou Bai

**Affiliations:** 1 Department of Tumor Center, The First Hospital of Jilin University, Changchun, Jilin, China; 2 Department of Laboratory Medicine, The First Hospital of Jilin University, Changchun, Jilin, China; H. Lee Moffitt Cancer Center & Research Institute, UNITED STATES

## Abstract

**Objectives:**

This study aimed to determine the HBV infection status of 135 patients with DLBCL (diffuse large B cell lymphoma), to analyze the overall survival (OS) and progression-free survival (PFS) of the different HBV infection status groups, and to discuss the relationship between HBV serological test results and the prognosis of DLBCL patients.

**Methods:**

A retrospective analysis was performed of the clinical data, HBV serological test results, and PFS/OS of 135 DLBCL patients who were initially diagnosed and treated with more than 3 cycles of an R-CHOP/CHOP/CHOP-like regimen at our center from January 1, 2008 to December 31, 2012.

**Results:**

The patients in the HBV infection group were older at disease onset (≥60 years old) and were more likely to present with stage 3-4 disease compared with the HBV-free group (P = 0.030 and P = 0.025, respectively). Approximately 50% of the patients with an active HBV infection required a reduction in the chemotherapy dose, and 66.7% of the patients in this group received more than 1 line of therapy; these rates were significantly higher than those in the no infection group (P = 0.003 and P = 0.011, respectively). Although HBV infection had no obvious influence on the outcome of first-line therapy, patients with an inactive infection had a higher relapse/progression rate within 3 months after a CR/PR than patients with an active infection (14/20 vs. 1/12, P = 0.001). The PFS at 1 year, 3 years and OS rates at 1 year, 3 years were significantly lower in the active HBV infection group than in the HBV-free group (P = 0.008, P = 0.002, P = 0.004, and P = 0.002, respectively). The PFS rates at 1 year and 3 year in HBV-free group were higher than those in the HBV infection group (80.5% and 52.9% P = 0.001, 78.1% and 44.4% P = 0.002). The lymphoma-related mortality rates were 2.7% in the no infection group, 19.2% in the HBV infection group (P = 0.004), and 28.6% in the active HBV infection group (P = 0.001). Among the patients treated with MabThera, the PFS in the HBV infection group was 11 months in the HBV infection group and 67 months in the infection-free group (P = 0.000). A Cox regression model of PFS revealed that age ≥60 years and HBV infection were independent prognostic factors (age: P = 0.019, HR = 2.002, 95% CI 1.123-3.567; HBV infection: P = 0.026, HR = 0.494, 95% CI 0.265-0.919).

**Conclusion:**

Compared with the patients in HBV-free group, those in the HBV infection group were older at disease onset, and the active infection patients presented with more advanced disease and had a lower PFS at 1, 3 years as well as a lower OS at 3 years. The patients in the inactive infection group had a higher progression/relapse rate within 3 months after a CR/PR than those in the active infection group. HBV infection was an unfavorable factor for PFS in the MabThera group. An age ≥60 years and HBV infection were independent unfavorable prognostic factors for PFS.

## Introduction

In the era of MabThera, the cure rate for DLBCL has increased significantly beyond that for the standard therapeutic strategy, but conventional treatment is still ineffective in approximately 30% of patients [1]; the reasons for this lack of activity in certain patients require investigation. HBsAg+ rate was 12%-30% in NHL patients, and about 25%-61% in DLBCL. The rate was higher than the general population [2–6]. R-CHOP or CHOP/CHOP-like regimens are the standard first-line therapy for DLBCL patients [7–8]. However, there are certain disadvantages to first-line chemotherapy, including HBV reactivation in response to MabThera and activated or aggravated HBV infection status [9–11]. Therefore, a timely and effective cure is difficult to achieve in patients infected with HBV. The data seem to indicate that this group of patients may have a worse therapeutic outcome. In China, HBV is present in more than 100 million people, and the HBsAg-positive rate is 9.09% [12–13]. HBsAg positivity is an unfavorable prognostic factor for patients treated with MabThera [14], but whether patient`s prognosis is influenced by the presence of an HBV infection has not been reported. This article reviewed complete clinical profiles of 135 patients who were newly diagnosed with DLBCL-nos and hospitalized in the First Hospital of Jilin University during the 5 years (2008.1–2012.12).This study sought to determine whether HBV infection influenced the prognosis of patients with DLBCL and to analyze possible reasons for this effect.

## Data and Methods

### 1.1 General information

222 Patients who initially diagnosed with DLBCL-NOS between January 1, 2008 and December 31, 2012 who hospitalized in the First Hospital of Jilin University were reviewed. 145 patients had complete clinical files. 135 of them were included because accepted the first line therapy as R-CHOP or CHOP/CHOP-like and agreed to be followed up. All the methods utilized in this study were approved by the ethics committee of the First Hospital of Jilin University, and the research was conducted according to the principles expressed in the Declaration of Helsinki. Written or oral informed consent was obtained from the 135 participants: 102 individuals provided written informed consent, and 20 individuals provided oral consent because they lived far from our hospital. The oral consents were recorded as stipulated by the approval from the ethics committee. As approved by the ethics committee, portions of the data on the 13 individuals who were lost to follow-up are described in this manuscript, analyzed as censored data.

Patients who refused treatment or who refused first-line regimens were excluded. All the patients underwent more than 3 cycles of the regimen described above, MabThera, doxorubicin, cyclophosphamide, vincristine, prednisone. The final cohort included 135 patients, aged 28–89 years, with a median age of 59 years. Overall, 74 patients were male, 61 were female, and the male: female ratio was 1.21:1.

All the cases were strictly diagnosed based on a biopsy according to the lymphatic hematopoietic system malignant tumor classification standard (WHO, 2008) [15]. All the cases were clinically staged according to the Ann Arbor staging criteria [16]: 18 cases were stage I (13.3%), 30 cases were stage II (22.2%), 43 cases were stage III (31.9%), and 44 cases were stage IV (32.6%). The normal LDH range was 135–226 U/L based on our hospital’s lab standard, and 51 patients (37.8%) exhibited increased LDH levels. According to the immunohistochemical results, the Hans algorithm [17] yielded 74 GCB cases (54.8%) and 61 non-GCB cases (45.2%).

HBV serum from 135 patients (100%) [18] were screened using the chemiluminescence method. The test contains five items, HBsAg, HBsAb(anti-HBs), HBeAg, HBeAb(anti-HBe), HBcAb(anti-HBc).The patients’ serological results were classified as follows: 1, HBV-free group: HBsAb+ only or all items negative; 83 patients (61.5%); means previous immunization or never infected by HBV. 2, others: called HBV-infection group: they had any combination of HBsAg+, HBeAb+, HBeAg+, HBcAb+ or HBsAb+; 52 patients (38.5%), means who ever infected by HBV, including all the possible stage during HBV infection. In this HBV infection group, the age of onset ranged from 32–88 years, and the median age was 61 years. Within this group, 31 patients were male, 21 were female, and the male-female ratio was 1.48:1. Of the patients with an HBV infection, 34 were positive for HBV DNA (33/52, 65.4%) in tests using the fluorescent quantitative PCR method (normal range: <500). 3. Active HBV infection group (a sub-group of group 2) was comprised of individuals who were HBsAg+ and/or HBcAb+(anti-HBc) combined with exhibited increased HBV DNA and/or ALT evaluated >2 UNL, means infectious status. This group consisted of 21 patients (15.6%); the age of onset ranged from 32–75 years, and the median age was 58 years. In this group, 13 patients were male, 8 were female, and the male-female ratio was 1.63:1. (Table 1)

**Table 1:** General information.

	HBV-free group (n = 83)	HBV-infection group (n = 52)	Active HBV infection group (n = 21)	P-value
**Age**				
≤60	54 (65.1%)	24 (46.2%)	12 (57.1%)	0.030*
>60	29 (34.9%)	28 (53.8%)	9 (42.9%)	0.501**
**Sex**				
Male	45 (54.2%)	31 (59.6%)	13 (61.9%)	0.490*
Female	39 (45.8%)	21 (40.4%)	8 (38.1%)	0.492**
**Stage**				
I-II	33 (39.8%)	11 (21.2%)	5 (23.8%)	0.025*
III-IV	50 (60.2%)	41 (78.8%)	16 (76.2%)	0.175**
**IPI (aaIPI)**				
≤2	62 (74.7%)	32 (61.5%)	16 (76.2%)	0.106*
>2	21 (25.3%)	20 (38.5%)	5 (23.8%)	
**LDH**				
Normal	55 (66.3%)	28 (53.8%)	9 (42.9%)	0.049**
Increased	28 (33.7%)	24 (46.2%)	12 (57.1%)	
**IHC**				
GCB	46 (55.4%)	28 (53.8%)	8 (38.1%)	0.156**
Non-GCB	37 (45.6%)	24 (46.2%)	13 (61.9%)	

*the HBV-free group compared with the infection group,

**the HBV-free group compared with the active infection group.

### 1.2 Treatment

All the 135 patients received the first-line treatment regimen—R-CHOP/CHOP/CHOP-like, 28 patients abandoned Mab Thera because of individual reason (economical reason mostly) (20 in HBV-free group and 8 in HBV infection group); 100 patients received MabThera (74.1%), and 16 underwent radiotherapy. The chemotherapy regimens (CHOP/CHOP-like)were administered in cycles of 21 days, the median courses was 6 (range 3–8). In active HBV infection group, at the very beginning of the therapy time, 66.7% (14/21) patients could not receive MabThera, 57.1%(12/21) were forced to reduce the chemotherapy dose, 23.8%(5/21) avoided MabThera and reduced the chemotherapy dose, and 33.3% (7/21) patients received radiation therapy, all of these were blamed for HBV activation. All 21 patients with active HBV infection received antiviral therapy (lamivudine and entecavir) for at least 6 months after the end of chemotherapy [19]. Among these patients, 6 was forced to delay chemotherapy until the HBV DNA content decreased to 10^5^ /ml and ALT fell to normal range as well. 20 patients whose HBV DNA level and ALT were normal but HBV serological detection were abnormal (12 was HBsAg+HBcAb+HBeAb+, and 8 were HBsAg+HBcAb+HBeAg+), they also received preventive entecavir treatment. All the patients with HBV infection were analyzed for HBV serum test and the active infection patients were analyzed HBV DNA content before every cycle and all the patients needed to underwent regular analyses of blood and liver (especially ALT) and kidney function (Table 2) [20, 21].

**Table 2:** Treatment.

	HBV-FREE group (n = 83)	HBV infection group (n = 52)	Active HBV infection group (n = 21)	P-value
**Stopped R**	20 (24.1%)	15 (28.8%)	7 (33.3%)	0.388
**Chemotherapy dose reduced**	17 (20.5%)	13 (25.0%)	11 (52.4%)	0.003
**Add RT**	8 (9.6%)	7 (13.2%)	4 (19.0%)	0.256
**Lines of chemotherapy >1**	30 (36.1%)	24 (46.2%)	14 (66.7%)	0.011

R: MabThera. RT: radiation therapy. P-values: the HBV-free group compared with the active infection group

### 1.3 Follow-up

From the time of diagnosis, all the cases were followed-up by telephone, and the deadline for follow-up was March 31, 2015. 15 cases were lost to follow-up, and the follow-up rate was 88.9%. The median follow-up time was 40 months, and the patients were followed-up for at least 6 months after the end of first-line therapy. The data from patients who died of an illness or accident unrelated to lymphoma, who failed to complete follow-up, or whose relatives could not provide follow-up information were excluded from survival analyze.

### 1.4 Statistical analysis

SPSS 20.0 software was used for the statistical analyses. Survival was analyzed using life tables and the Kaplan-Meier method. The single factor analyses were performed using the Log-rank test, and the general and treatment data were compared using the Χ^2^ test. P ≤0.05 signified statistical significance.

## Results

### 2.1 General information

The HBV infection rate among all the DLBCL patients was 38.5% (52/135). The active HBV infection rate was 15.6% (21/131). The HBV infection group was significantly older than the HBV-free group (53.8% vs. 34.9%, P = 0.030). Of the patients in the HBV infection group, 78.8% presented with stage III-IV disease; this rate was significantly higher than that for patients in the no infection group (60.2%) (P = 0.025). The proportion of LDH increased patients in active group was higher than the no infection group (57.1% vs. 33.7%, P = 0.049). (Table 1)

### 2.2 Treatment

In the HBV infection group, 26.9% (14/52) of the patients avoided MabThera, and 13.5% received combination radiotherapy (7/52). More than half (12/21) of the active HBV infection patients required a reduction in the chemotherapy dose; this rate was significantly higher than that for the HBV-free group (52.4% vs. 20.5%, P = 0.003). More patients with an active HBV infection received more than one line of chemotherapy compared with patients without HBV infection (66.7% vs. 36.1%, P = 0.011). (Table 2)

### 2.3 Curative effect

The 2007 Response Criteria for Malignant Lymphoma standard was used to assess the curative effects after first-line treatment [16]: 44 patients exhibited CR (32.6%), 50 had PR (37.0%), 9 exhibited SD (6.7%), and 31 experienced PD (22.8%). The ORR was 69.6% (Table 3). For the CR/PR patients, the relapse/progression rate within 3 months was 4.8% (3/62) in HBV-free group, 46.9% (15/32) in the HBV infection group and 8.3% (1/12) in the active HBV infection group. The HBV infection group was divided based on HBV infection status (active infection vs. other status), and recent disease progression (relapse/progression within 3 months) occurred significantly less frequently in the active HBV infection subgroup compared with the other infection subgroup (1/12(8.3%) vs. 14/20(70%), P = 0.001). Throughout the treatment process, 4 patients experienced an HBV reactivation (according to the expert consensus on the management of lymphoma with HBV infection: HBV reactivation was defined as ①when HBsAg+, HBV-DNA remeasurable from unpredictable, or HBV-DNA rise a logarithmic compared with the baseline, or HBeAg re-posotive; ②when HBsAg-/HBcAb+, HBsAg re-positive or HBV-DNA remeasurable from unpredictable). All of the 4 patients were HBsAg+ with HBV-DNA elevating when diagnosed. All reactivations occurred in the third or fourth cycles, and the treatment cycles were intermittently extended for all the reactivation patients due to antiviral treatment. One of these 4 patients died due to disease progression, whereas the other 3 patients maintained their PR status under regular treatment.

**Table 3:** Curative effect of first-line treatment.

	HBV-free group (n = 83)	HBV infection group (n = 52)	Active HBV infection group (n = 21)	P-value
**CR**	29 (34.9%)	15 (28.8%)	7 (33.3%)	0.462*
**PR**	33 (39.8%)	17 (32.7%)	5 (23.8%)	0.175**
**SD**	5 (6.0%)	4 (7.7%)	2 (9.5%)	0.627**
**PD**	15 (18.1%)	16 (30.8%)	7 (33.3%)	0.126**

*the HBV-free group compared with the infection group,

**the HBV-free group compared with the active infection group.

### 2.4 Survival

Overall, the OS was 94.0% (125/133) at 1 year, 84.2% (80/95) at 3 years, and 38.5% (20/52) at ≥5 years. The median PFS was 22.15months, and the median OS was 81.03 months. The average number of chemotherapy regimens was 1.78 in the HBV infection group and 1.3 in the HBV-free group. The 1year and 3year OS was 97.6% and 90.6% in the HBV-free group, which was significantly higher than the 76.2% and 54.5% in the active HBV infection group (P = 0.004, 0.002). The 1 and 3year PFS rates in the HBV-free group were 80.5% and 78.1%; these rates were higher than those observed in the HBV infection group (52.9% and 44.4%; P = 0.001 and P = 0.008, respectively) and in the active HBV infection group (52.4% and 27.3%; P = 0.002 and P = 0.002, respectively). The median follow-up was 40 months. During the study period, 26 patients died (20.8%), of which 8 did not have regular checkups, and the cause of death was unclear. The other 18 patients died of disease progression. Mortality was lower in the HBV-free group compared with the HBV infection and active HBV infection groups (2.7%, 19.2%, and 28.6%, respectively; P = 0.004, 0.001). Comparisons between the groups are shown in Table 4.

**Table 4:** Survival.

	HBV-free group	HBV infection group	Active HBV infection group	P-value
**OS**				
**1 year**	80/82	45/51	16/21	0.054*, 0.004**
**3 years**	58/64	22/27	6/11	0.222*, 0.002**
**5years**	10/14	10/12	2/4	0.652*, 0.569**
**PFS**				
**1 year**	66/82	27/51	11/21	0.001*, 0.008**
**3 years**	50/64	12/27	3/11	0.002*, 0.002**
**5 years**	9/14	6/12	0/4	0.462*, 0.082**
**Death**	2/73 (2.7%)	10/52 (19.2%)	6/21 (28.6%)	0.004*, 0.001**

* the HBV-free group compared with the infection group,

** the HBV-free group compared with the active infection group.

### 2.5 Single variable analysis

The median PFS was 18 months for patients with stage III-IV disease and 34 months for patients with stage I-II disease (P = 0.009); the median OS was 59 months for patients with stage III-IV disease but was not available for patients with stage I-II disease (P = 0.016). In patients aged ≥60 years compared with those aged <60 years, the respective median PFS times were 14 months and 67 months (P = 0.003), and the respective median OS times were 52 months and 57 months (P = 0.205). The median PFS for the patients with increased LDH levels was 12 months compared with 67 months for those with normal levels (P = 0.001), and the median OS values were 50 months (increased LDH) and not available (normal LDH) (P = 0.004). For patients with IPI (aaIPI) >2 compared with those with aaIPI ≤2, the respective median PFS values were 11 months and 67 months (P = 0.000), and the respective median OS values were 17 months and not available (P = 0.000). These data confirmed that stage III or IV disease, an age greater than 60 years, IPI/aaIPI 3-4/5 score and increased LDH levels were independent factors for negative prognosis.

**Fig 1:**
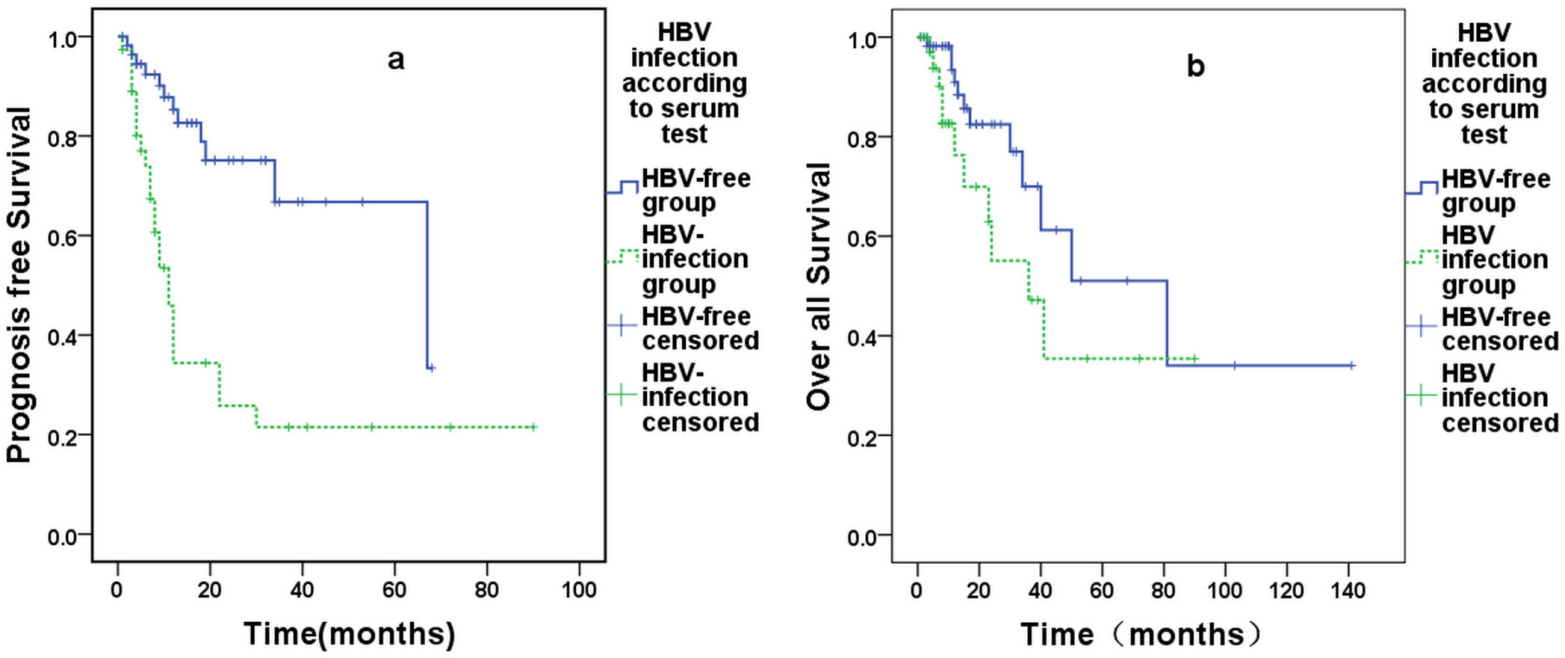
MabThera-treated 99 patients, 61 in HBV-free group (solid line) and 37 in HBV infection group (dotted line). (**A)**showed the PFS of the two group, the estimated PFS of HBV-free group was 67months, and HBV infection group was 11 months, P = 0.000. (**B)**showed the OS of the two group the estimated result of the HBV-free and infection group as 81months and 36months, P = 0.102.

**Fig 2:**
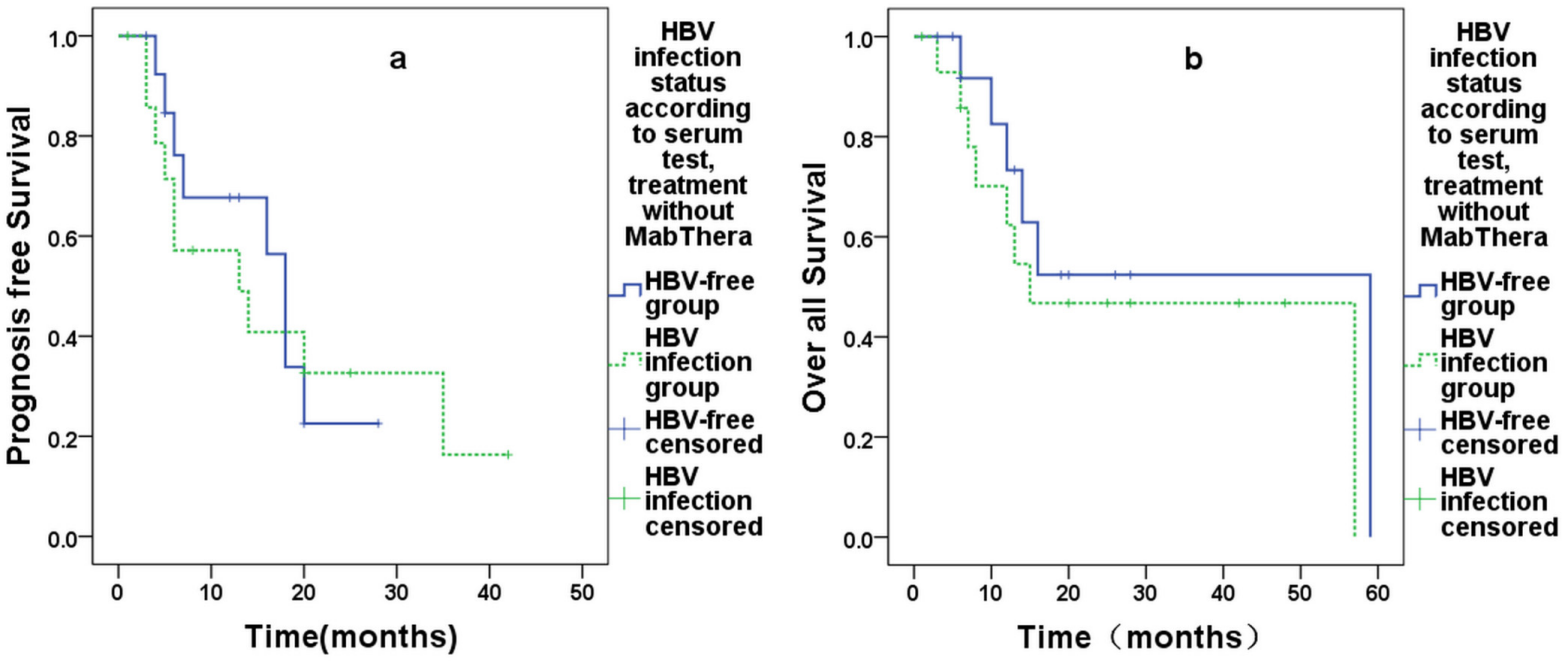
MabThera-treated 36 patients, 19 in HBV-free group (solid line) and 27 in HBV infection group (dotted line). **(A)**showed the PFS of the two group, the estimated PFS of HBV-free group was 59 months, and HBV infection group was 15 months, P = 0.426. **(B)**showed the OS of the two group the estimated result of the HBV-free and infection group as 18 months and 13months, P = 0.837.

The influence of MabThera on survival was assessed. After receiving MabThera, the HBV infection group had a significantly lower PFS than the HBV-free group, with estimated PFS values of 11 months and 67 months, respectively (P = 0.000, Fig 1A), and OS values of 36 months and 81 months, respectively (P = 0.102, Fig 1B). Without MabThera treatment, there were no significant differences in OS or PFS in the HBV infection group (PFS: 15 months vs. 59 months, P = 0.426; OS: 13 months vs. 18 months, P = 0.837) (Fig 2A and 2B).

### 2.6 Multi-variate analysis

Multi-variate factors, such as age, LDH levels, stage, IPI/aaIPI and HBV infection status (abnormal HBV serum test), were analyzed by Cox regression analysis (Table 5). Age ≥60 years and HBV infection were negative prognostic factors for DLBCL; the respective HRs were 2.002 (95% CI 1.123–3.567; P = 0.019) and 0.494 (95% CI 0.265–0.919; P = 0.026). (Table 5)

**Table 5:** Multi-factor analysis of DLBCL.

	B	SE	Wald	Sig	HR	95.0%CI of HR	
						lower	upper
**age ≥60**	0.694	0.295	5.464	0.019	2.002	1.123	3.567
**stage 3–4**	-0.380	0.435	2.320	0.383	0.684	0.291	1.606
**increased LDH**	-0.212	0.331	1.829	0.521	0.809	0.423	1.546
**IPI/aaIPI 3-4/5**	-0.632	0.338	3.493	0.062	0.531	0.274	1.031
**HBV infection**	-0.705	0.320	6.130	0.026	0.494	0.265	0.919

## Discussion

In our DLBCL cohort, the HBV infection rate was 38.5%, the active HBV infection rate was 15.6% (HBsAg-positive rate, 13.3%), and both of these values were higher than the previously published HBV infection rate (30–35%) and HBsAg-positive rate (9%) from epidemiological studies in China. Higher HBV infection rates in lymphoma patients have been reported [2–4, 6], and our results are in accordance with these values. Age, disease stage and IPI/aaIPI LDH are poor prognostic factors for DLBCL, and our data revealed that HBV-infected patients were older at disease onset and that these patients presented with higher IPI/aaIPI and more advanced disease, which suggests that patients with HBV infection have a worse prognosis.

MabThera increases the risk of HBV reactivation, depletion of B cells in the circulation is the main mechanism, and this may induce immune disorder toward HBV. So not only HBsAg+ but HBsAg-/HBcAb+ status also may be reactivated. Therefore, the use of MabThera requires careful observation, and the HBV DNA content must be monitored closely. In our cohort, more than a quarter of the HBV-infected DLBCL patients (28.8%) could not be treated with MabThera, and 13.3% of these patients received radiotherapy along with standard chemotherapy with the intention of waiting for improved HBV DNA levels. Those with an active HBV infection faced more challenges, as half of them were forced to reduce their chemotherapy dose. These data suggested that once an individual has been infected with HBV, the therapeutic effect will be smaller than that for those without an infection, particularly compared to those with an active infection, even though the curative effect has not yet been evaluated. The statistical analysis revealed that the patients in the active infection group received an average of 1.78 chemotherapy regimens, which was significantly more than the number received by the no infection group (1.3).

The subsequent survival analysis confirmed our hypothesis. The active HBV infection group had a significantly lower both OS and PFS of 1-year, and 3-year as well, than the HBV-free group (76.2% vs. 97.6%, 54.5% vs. 90.6%, 52.9% vs. 80.5% and 44.4%vs. 78.1%, respectively). The 1-year and 3-year PFS values were lower in the HBV infection group than the no infection group. Although 5 year data did not suggest significant differences, consider associated with the unsufficient cases number. This suggested that HBV infection may confer a worse prognosis for patients with DLBCL. It is possible that this result was influenced by the reduced chemotherapy dosage or the lack of MabThera treatment. Therefore, we separated the patients based on the presence or absence of MabThera treatment and re-analyzed the survival rates. The median PFS of the HBV-infected patients treated with MabThera was 11 months, but the median PFS was 67 months for non-infected patients (P = 0.000). Certainly, there were patients with a high HBV viral load, and their therapy was slightly delayed compared with the other patients; therapy began once the HBV DNA level decreased to 10^5^, and these patients all regularly received MabThera for at least 3 cycles. There were no differences in median PFS for patients without MabThera compared with non-infected patients. In the era of MabThera, HBV infection has important effects on the prognosis of patients with DLBCL, and similar results have been previously reported [22–23].

The median PFS was 22.15 months for all the DLBCL patients, 34.21 months for the patients on MabThera and 16.58 months for those not receiving MabThera treatment. Therefore, the use of MabThera increased the overall therapeutic effect in patients with DLBCL [1,7–8]. However, whether the HBV infection was the unique factor that influenced the change in PFS from 34.21 months to 11 months could not be confirmed by this study [24]. The HBV-infected patients received lower chemotherapy dosages, and a large percentage of these patients could not take MabThera; therefore, the treatment progress was more variable. Future studies will include detailed stratified analyses of different therapeutic intensities. Additionally, among the HBV-infected patients, the relapse/progression rate 3 months after the first CR/PR differed among those with a different infection status: the rate in patients with an active infection was markedly lower than that in patients with other infection statuses (1/12 vs. 14/20, P = 0.001). We cannot fully explain this result; however, we suspect that this result is related to taking greater precautions with patients with an active infection [25]. Our Cox analysis showed that HBV infection (abnormal HBV serum test result) was a negative prognostic factor for DLBCL. This finding renewed interest in HBV-infected patients who were not only HBsAg+ or HBV-DNA elevated; thus, future studies will analyze the relationship between the different HBV infection statuses and the prognosis for patients with DLBCL.

In the aspect of antiviral treatment, though there is still no global systematic consensus about the anti-HBV in B cell lymphoma, our center refered to the “chronic HBV prevention and control guidelines”. All the 21 active infection patients accepted antiviral treatment in our center 16 of them took entecavir, and other 5 took lamivudine. And 20 of the rest 31 patients in infection group, accepted entecavir preventive treatment (took antiviral drug from beginning to at least 6 months from end of regular therapy). As we observation, no one was influenced the lymphoma treatment because of HBV reactivation in the 20 patients, and 4 patients had transient ALT elevating. This result conformed to the report from Huang H et al [26], but If this could be replicated need more large-scale multi-center clinical research.

## Conclusion

The patients with an active HBV infection were older, presented with more advanced disease, experienced a worse therapeutic result, and displayed higher relapse rates. Even with more attention on antiviral treatments, there has been little progress in reducing the progression of DLBCL in patients infected with HBV. HBV infection may become an independent negative prognostic factor for DLBCL patients. Active infection of patients with DLBCL need high attention, and serology prompt the not active stage of infection of patients with HBV patients also need to get attention. First, close monitoring of HBV serological results, combining with ALT, HBV DNA, second if the economic conditions allowed and well-compliance in the patients, accepted preventive treatment can yet be regarded as one of the effective methods. A wider study on large number of patients is recommended.
